# Dietary Schizochytrium Microalgae Affect the Fatty Acid Profile of Goat Milk: Quantification of Docosahexaenoic Acid (DHA) and Its Distribution at Sn-2 Position

**DOI:** 10.3390/foods11142087

**Published:** 2022-07-14

**Authors:** Huiquan Zhu, Xiaodan Wang, Wenyuan Zhang, Yumeng Zhang, Shuwen Zhang, Xiaoyang Pang, Jing Lu, Jiaping Lv

**Affiliations:** Institute of Food Science and Technology, Chinese Academy of Agricultural Sciences, No. 1 South Road, Malianwa, Haidian District, Beijing 100193, China; 13834083105@163.com (H.Z.); prettyshoot@126.com (X.W.); zwy7183425@163.com (W.Z.); zym18811735237@hotmail.com (Y.Z.); 15811499853@163.com (S.Z.); pangxiaoyang@163.com (X.P.); lujing@caas.cn (J.L.)

**Keywords:** microalgae, DHA, absolute concentration, sn-2 fatty acids, goat milk

## Abstract

The objective of this study was to detect the influence of dietary Schizochytrium microalgae on milk composition, milk fatty acids, and milk sn-2 fatty acids in goat’s milk. Firstly, we could see that the fat content increased in low microalgae supplementation goat’s milk (LM, 15 g/day) and the lactose content decreased in medium microalgae supplementation goat’s milk (MM, 25 g/day) compared with control goat’s milk (C, 0 g/day). Moreover, the absolute concentration of the docosahexaenoic acid (DHA) of LM, MM, and high microalgae supplementation (HM, 35 g/day) goat’s milk was 29.485, 32.351, and 24.817 mg/100 g raw milk, respectively, which were all higher than that in the control goat’s milk with 4.668 mg/100 g raw milk. In addition, the sn-2 DHA content increased in MM and HM goat’s milk. However, the decreasing trend of the sn-2 DHA content was observed in LM goat’s milk. As for other fatty acids, the oleic acid (C18:1n9c) and linolenic acid (C18:3n3) content decreased and increased, respectively, in all experimental goat milk. Finally, an interesting phenomenon was found, which was that docosanoic acid (C22:0) and tetracosenic acid (C24:1) were only detected in test goat’s milk. Consequently, the phenomena of this study demonstrated that dietary Schizochytrium microalgae have an obvious effect on the fatty acid and sn-2 fatty acid profile of goat’s milk, and they provide an effective method to improve the content of goat’s milk DHA in practical production.

## 1. Introduction

Recently, due to the high-quality biomass property of microalgae, it is widely used in bio-energy, pharmaceuticals, food, feed industries, etc. Among all microalgae, Schizochytrium microalgae is rich in nutrients, such as protein, fat, sugar, and other nutrients. Moreover, the lipid content of Schizochytrium microalgae is higher than 70% in comparison with other microalgae. Triglyceride is the main form of Schizochytrium microalgae oil, which took up about 90% of the total lipids [1]. In addition, the fatty acid (FA) content of Schizochytrium microalgae accounted for 18.3~25% of its dry matter weight. The FA profile of Schizophytrium microalgae is abundant, which is rich in unsaturated fatty acids (UFA) and mainly composed of DHA, docosapentaenoic acid (DPA), and palmitic acid (C16:0) [2,3]. A previous study reported that the DHA content of total fatty acids is 35% [4].

DHA plays a vital role in the brain health, growth, and development of infants [5]. Jensen (2005) reported the addition of DHA to a pregnant women’s diet could improve the cognitive ability of infants [6]. Moreover, metabolic abnormalities controlled by the fetal central nervous system were caused by the severe deficiency of DHA for the fetus [7]. Birch, E.E. (1998) reported that the visual acuity of infants was improved significantly by continuous feeding of DHA-rich foods [8]. However, the desaturase activity of infants was extremely low and could not satisfy the requirements of DHA synthesis [9]. Therefore, more and more DHA-enhanced infant formula milk powders, which were directly added with microalgae or fish oil, were produced in China, Japan, and the United States to satisfy the daily nutrient requirement of infants [7,10].

However, ruminant milk directly enriched with algal oil may result in a fishy flavor, leading to the unacceptance of consumers. Thus, microalgae had been registered as animal feed additives to increase the content of omega 3 fatty acids in ruminant milk [11]. Microalgae had been widely studied to increase the UFAs content of cow’s milk in the last two decades. Franklin (1999), Vahmani (2013), and Liu (2020) reported that the content of DHA in cow’s milk increased significantly after dietary microalgae, with the increasing ratio of DHA going from 100% to 760% [12,13,14]. In addition, the increasing trend of DHA content was observed in dairy goat’s milk [15,16]. Undoubtedly, ruminant milk fat exists as the triglyceride (TAG) form, which is constituted of three fatty acids and one glycerin [17]. When the dietary fat enters the rumen, the fatty acid of TAG is hydrolyzed by lipase, and then the free fatty acid was mainly hydrogenated and produced by the microorganism [18].

The use of Schizochytrium microalgae in dairy goat fodder has rarely been reported. Moreover, the content of DHA in Schizachyrium microalgae is higher compared with other DHA-rich sources, and the direct feeding with Schizachyrium microalgae can reduce production costs in comparison to the addition of microalgae or fish oil in goat’s fodder. Hence, the milk composition, milk fatty acids, and milk sn-2 fatty acids of goat’s milk were detected after the supplement of Schizochytrium microalgae into Shanxi Guanzhong goats’ daily diet in this study, and the results provide theoretical support for producing high-DHA goat’s milk.

## 2. Materials and Methods

### 2.1. Materials

The Schizochytrium microalgae powder was provided by Xiamen Huison Biotech Co. Ltd. The tridecylic acid triglyceride (C13:0 TAG) and porcine pancreatic lipase (type II) were purchased from Beijing Manhage Biotechnology company and Sigma Aldrich (St. Louis, MO, USA), respectively. Trichloromethane, diethyl ether, petroleum ether, Sodium cholate hydrate (65%), and potassium hydroxide were bought from Sino Pharm Chemical Reagent (Shanghai, China). Methanol (>99.9% purity) and *n*-Hexane (≥98% purity, chromatographic grade) were obtained from Aladdin (Beijing, China). Moreover, 37 fatty acid methyl esters (FAMEs) were purchased from ANPEL Laboratory Technologies Inc. (Shanghai, China). All other solvents and reagents were analytical grade (Sino Pharm Chemical Reagent, Shanghai, China).

### 2.2. Sample Collection and the Detection of Milk Composition

All animal procedures in this experiment were approved by the Animal Care and Use Committee of the Institute of Food Science and Technology, Chinese Academy of Agricultural Sciences (IFST-2019-105), and conducted in keeping with animal welfare and ethics. Specifically, 120 Guanzhong dairy goats, who had been pregnant twice and were in middle lactation stage, were randomly divided into four groups, including the control (C, 0 g/day), low microalgae supplementation (LM, 15 g/day), medium microalgae supplementation (MM, 25 g/day), and high microalgae supplementation (HM, 35 g/day) groups. To ensure the microalgae was adequately eaten, the microalgae powder was mixed thoroughly by a machine. In addition, dairy goats were reared in a specific barn and allowed to freely drink water, and they were milked once a day at three o’clock in the morning. The experiment period lasted 65 days, containing a 15-day adaptation period. Diet formula and fatty acids of microalgae powder are shown in Table 1 and Table 2, respectively.

The goat’s milk was obtained at the end of the experiment. Afterward, these milk samples were cooled to 4 °C and transported from Xi’an to Beijing Lab on the same day, and then separated into two parts. One part was stored in the refrigerator at −80 °C for fatty acids and sn-2 fatty acids analysis. The other part was added to a tube and detected by a milk component detector for milk composition at Beijing Dairy Cow Testing Center.

### 2.3. Determination of Fatty Acids Profiles

The detection method for fatty acid (FA) was referred to in a previous study and modified slightly [19]. To be specific, 250 μL goat milk, 300 μL internal standard solution (C13:0 TAG, 3,1 mg/mL), 2 mL methanol, 2 mL HCL/methanol (3N), and 1 mL *n*-hexane were mixed and vortexed in screw glass test tube. In the next step, the tube was tightly capped and then heated for 1 h at 100 °C in the water bath. After that, the tube was cooled to room temperature, 2 mL water was pipetted into it, and it was vortexed for 30 s. Finally, this tube was centrifuged at 1200× *g* for 5 min, and the upper phase (*n*-hexane) was transferred and filtered through a 0.22-µm filter into the vials for the gas chromatography (GC) test.

The FAMEs sample was analyzed by GC with a hydrogen flame ionization detector (Agilent 8890 B) and a capillary column (DB-23 60 m × 0.25 mm × 0.25 μm; Sigma-Aldrich). Both the injector and detector temperatures were 250 °C. Nitrogen was used as the carrier gas with a flow rate of 0.8 mL/min and the split ratio was 1:20. The program of the column oven temperature was as follows: the initial temperature was 50 °C and kept for 1 min, and then the column oven temperature increased to 175 °C with a rate of 20 °C/min. Afterward, the column oven was heated to 230 °C at a rate of 1.3 °C/min and maintained for 5 min. The FAMEs were identified by the retention times comparison between the sample peaks and those of known FAME standards.

Quantification of DHA was performed using the following formulas and data are expressed in mg of fatty acid per 100 g of goat milk:

$$X_{\mathrm{DHA}}{=F}_{\mathrm{DHA}}\;\times \;\frac{A_{\mathrm{DHA}}}{A_{C13}}\;\times \;\frac{M_{C13}\;\times \;1.0059}{M}\;\times \;0.9590\;\times \;100\;\times \;1000\;F_{\mathrm{DHA}}=\frac{\rho_{\mathrm{FAMEs}-\mathrm{DHA}}{\;\times \;A}_{\mathrm{FAMEs}-C13}}{A_{\mathrm{FAMEs}-\mathrm{DHA}}{\;\times \;\rho }_{\mathrm{FAMEs}-C13}}$$

where X_DHA_: quantity of DHA expressed as mg per 100 g goat milk; FDHA: response factor; A_DHA_: peak area of DHA in the sample chromatogram; A_C13_: peak area of C13:0 internal standard in the sample chromatogram; M_C13_: mass of C13:0 internal standard added to the sample solution, in mg; 1.0059: conversion coefficient of triglyceride to methyl of C13:0; M: mass of test sample, in mg; 0.9590: the coefficient of DHA fatty acid methyl ester converted into fatty acid; 1000: the conversion coefficient between grams and milligrams; ρ_FAMEs-DHA_: the concentration of DHA in the standard mixture of 37 FAMEs; A_FAMEs-C13_: peak area of C13:0 in the 37 FAMEs; A_FAMEs-DHA_: peak area of DHA in the 37 FAMEs; ρ_FAMEs-C13_: the concentration of C13:0 in the standard mixture of 37 FAMEs.

### 2.4. Detection for Sn-2 Fatty Acids

The milk fat was extracted following a modified version of the Folch method [20]. Briefly, 3 mL goat milk, 6 mL methanol, 12 mL trichloromethane, and 6 mL water were added to the tube, and then the mixed solution was vortexed thoroughly and centrifuged at 3500 rpm for 15 min. In the next step, the bottom solution was extracted and dried by nitrogen gas.

The detection method for sn-2 fatty acids included the preparation of sn-2 MAG and methylation, and it was referred to the previous study reported by Qi (2018) and Sahin (2005) [21,22]. Specifically, 200 μL *n*-hexane, 2 mL TRIS buffer (pH 8.0), 0.5 mL bile salts (0.05%), 2 mL calcium chloride (2.2%), and 20 mg pancreatic lipase (porcine pancreatic lipase type II) were pipetted into the tube which contained 25 mg milk fat. The mixture was hydrolyzed by shaking in a water bath (37 °C) for 40 min. After the hydrolysis, 1 mL HCl solution (6 M) and diethyl ether were added in sequence into the mixture solution. In the next step, the mixing solution was vortexed for 2 min and then centrifuged subsequently at 4000 rpm for 5 min. After that, the supernatant was transferred into a new tube and separated on a silica gel plate with a developing solvent that comprised *n*-hexane, diethyl ether, and acetic acid (50:50:1, *v*/*v*/*v*). Finally, the band corresponding to sn-2 MAG was isolated and extracted twice with 5 mL diethyl ether. The diethyl ether blended with sn-2 MAG was removed by nitrogen gas, and then it was methylated and detected by the GC method mentioned in 2.3 of this study.

### 2.5. Statistical Analysis

The percentage of fatty acid and sn-2 fatty acid is expressed as mean and pooled standard errors (SEM), and they were subjected to one-way analysis of variance using SPSS software (Version 24, SPSS, Chicago, IL, USA). The normality and homogeneity of variance were assessed by Shapiro–Wilk and Levene’s test, respectively. Bonferroni and Dunnett’s T posthoc tests were used to identify the difference when variance did or did not satisfy the condition of homogeneity.

## 3. Results

### 3.1. Quantification of DHA and Other Chemical Compositions in Goat Milk

The influence of different addition amounts of Schizochytrium microalgae on dry matter intake, milk production, milk composition, and DHA absolute concentration are shown in Table 3. The content of goat milk production, protein, somatic cell, and dry matter intake was not influenced by the microalgae supplementation. Moreover, the milk fat content increased by 20.90% in LM goat’s milk (*p* < 0.05) in comparison with the control goat’s milk, while it remained stable in MM and HM goat’s milk. The lactose content decreased by about 4.5% in MM goat’s milk (*p* < 0.05).

Regarding the absolute concentration of DHA, it was evident that the DHA absolute concentration in the test goat’s milk was higher than that in the control goat’s milk. More specifically, the absolute concentration of DHA in the control goat’s milk was 4.668 mg/100 g raw milk. With the increase of the microalgae addition, the DHA absolute concentration increased to 29.485, 32.351, and 24.817 mg/100 g in LM, MM, and HM goat’s milk, respectively. Meanwhile, the increase rates of DHA were 657%, 716%, and 553% in LM, MM, and HM goat’s milk, respectively.

### 3.2. Milk Fatty Acids Composition

The profile of the milk fatty acid was affected significantly by the dietary microalgae (Table 4). The content of caproic acid (C6:0), decanoic acid (C10:0), and lauric acid (C12:0) increased (*p* < 0.05) in all the test goat’s milk in comparison with the control goat’s milk. On the contrary, the percentage of octanoic acid (C8:0) decreased (*p* < 0.05). Furthermore, the content of some long-chain saturated fatty acids (LC-SFAs), such as heptadecanoic acid (C17:0), eicosanoic acid (C20:0), and tricosoic acid (C23:0), all rose in the test goat’s milk. However, the myristic acid (C14:0) and palmitic acid (C16:0) content showed a stable trend. Moreover, the proportion of stearic acid (C18:0) increased in LM and MM goat milk; by contrast, it was decreased in HM goat milk. In addition, unsaturated fatty acid (UFA) was apparently affected by the microalgae in comparison with SFA. Most UFAs, including pentadecenic acid (C15:1), trans vaccenic acid (C18:1n7t), linolenic acid (C18:3n3), eicosapentaenoic acid (EPA, C20:5n3), and DHA, all showed a growing tendency in the test goat’s milk, while the oleic acid (C18:1n9c) content and the ratio of omega 6/omega 3 (n6/n3) decreased. It should be mentioned that docosanoic acid (C22:0) and nervonic acid (C24:1) were only detected in the test goat milk, and the content of C22:0 and C24:1 both increased.

### 3.3. Milk Sn-2 Fatty Acid Composition

The percentage of sn-2 fatty acid calculated by the Peak area normalization method is shown in Table 5. Compared with the total FA, the goat milk sn-2 FA was similarly influenced by the addition of microalgae. The content of C8:0 decreased with the supplement of microalgae. However, the increasing tendency was observed in C10:0, C12:0, and the total MC-SFA. Moreover, the other SC-SFAs’ and MC-SFAs’ contents remained constant. In terms of LC-SFA and UFA, it was notable that the percentage of C18:1n7t, C20:0, C22:0, C20:5n3, and C23:0 all increased in the test goat milk compared to control goat milk. By contrast, there was a declined trend observed in the content of C17:1, C18:1n9c, C18:3n3, n6/n3, and LC-SFA. The content of sn-2 DHA in the control goat’s milk was 0.166%. The sn-2 DHA content increased to 0.354 and 0.429% in MM and HM goat milk, with an increasing rate of 113% and 158%, respectively. However, the sn-2 DHA content decreased to 0.104% in the LM goat milk, with a decreasing rate of 37%.

## 4. Discussion

### 4.1. Milk Chemical Compositions and Dry Matter Intake

After the supplementation of Schizochytrium microalgae to the goat’s daily diet, the fat content increased significantly in the LM goat’s milk in comparison with the control goat’s milk in this study, which was in accordance with previous studies [15,23]. However, other researchers had reported that the milk lipid content decreased after dietary microalgae supplementation [24,25]. Moreover, some studies showed that there were no remarkable changes in the milk fat content [14,26], and the same observation was also noticed in MM and HM goat milk in this study. As for the factors which could induce the decrease of milk fat, Toral (2010) reported that it might be caused by the increase in fat content in test fodder compared with the control diet [27]. Moreover, other researchers thought there were some inhibitors of lipid synthesis produced by trans-fatty acids in the rumen after the addition of microalgae [24,28].

In addition, the content of milk lactose only decreased in MM goat’s milk in comparison with the control goat’s milk, and this result was in accordance with previous research [23,29]. However, the milk lactose content of the LM and HM goat milk showed a stable trend, which was in line with control goats and other studies [15,30]. Moreover, it was evident that the content of milk protein, somatic cells, production, and dry matter intake were stable after the microalgae supplementation of the fodder; this result was also observed in previous studies [30,31,32,33]. By contrast, a reduction in milk protein production and dry matter intake content had been reported by other researchers [25,27,28], and it was likely due to the rise of some microalgae histidines which could limit the production of milk protein [26].

### 4.2. The Content and Absolute Concentration of DHA

DHA is the major constituent of the Schizochytrium microalgae FA profile. In this study, the absolute concentration and proportion of DHA increased apparently in all the test goat’s milk compared to the control goat’s milk. Briefly, the absolute concentration of DHA was 29.485, 32.350, and 24.817 mg/100 g raw milk in LM, MM, and HM goat milk, respectively. Meanwhile, the percentage of DHA in the total fatty acid was 0.623%, 0.759%, and 0.585% in LM, MM, and HM goat milk, respectively. To know the variation of DHA with the addition of microalgae into ruminant fodder, the results of some published studies are summarized in Table 6. It was clear that the DHA content was increased in all studies by dietary microalgae, and the increasing rate of DHA ranged from 100% to 1510%. Moreover, cows were the major studied animal, and the proportion of DHA in the total FA varied from 0.02% to 0.91% in cow’s milk after the addition of microalgae. As for goat’s and ewe’s milk, the DHA percentage varied from 0.04% to 1.15%, and the range of its variation was wider than that in cow’s milk. In addition, the absolute concentration of DHA was only reported in one previous study, which was 15.7 mg/100 g raw milk and less than that in this study. This difference might be caused by the different calculating methods and feeding time.

DHA synthesized in ruminant milk mainly came from three sources: synthesized by microbes in the rumen and intestine of ruminant, originated from endogenic alpha-linolenic acid, and transformed from the daily diet [18]. The major part of dietary DHA, accounting for 60~98%, was hydrogenated in the rumen [34,35], and then the remaining DHA flowed into the intestine. The content of DHA absorbed by the intestine was taken up by 70~100%, and then the unabsorbed dietary DHA was transported to ruminant organs through the blood [18,36]. However, the level of DHA absorbed by mammary gland cells was only 13~25% from the intestine [37]. Thus, the effective strategy to improve the DHA content in ruminant milk through the use of diet was to restrain the DHA hydrogenation of rumen.

### 4.3. Fatty Acid Profile of Goat Milk

In this study, the content of goat milk fatty acid changed significantly in the test goat’s milk. On one hand, the percentage of most SC-SFAs and MC-SFAs, such as C6:0, C10:0, and C12:0, increased in all the test goat’s milk, which was in accordance with the conclusions made by Pajor (2019) and Alexandros Mavrommatis (2020) [16,41]. However, other studies reported that the proportion of C6:0, C10:0, and C12:0 all decreased after the supplementation of microalgae in goat’s and cow’s fodder [14,15,24,38]. Moreover, as for LC-SFAs, the microalgae feeding did not influence the content of C14:0, C15:0, and C16:0, and this result was in line with the phenomenon reported by previous studies [16,30]. Other studies found the content of C14:0, C15:0, and C16:0 increased and decreased in goat’s and cow’s milk, respectively [13,28,37].

On the other hand, the proportion of C18:1n9c and C18:2n6c all decreased in the test goat’s milk compared with the control goat’s milk. Moreover, the C18:0, C18:1n7t, and C18:2n6t content demonstrated the rising tendency. This phenomenon was in accordance with the conclusion of previous research and the probable reason was that the microorganism hydrogenation in rumen was limited by the microalgae [11,22,27,37]. However, Eleni Tsiplakou (2017) reported that the content of fatty acids with 18 carbons was not influenced by the addition of microalgae [30]. Moate, P.J (2013) found that c18:2n6c in the cow’s milk after being fed microalgae was higher compared with the control cow’s milk [24]. The content of C20:5n3 (EPA), an omega 3 fatty acid that is beneficial to human health [42], increased after dietary microalgae supplementation, which is in line with the results obtained from the goat’s and cow’s milk [15,23,30,40]. However, Liu (2020) reported that the C20:5n3 content decreased in cow’s milk after these cows were fed with microalgae [14]. The ratio of n6/n3 is important for the synthesis of eicosanoid in human body, and it is regarded as a vital nutrition indicator of milk fat [16,43]. The inclusion of Schizochytrium microalgae into the goats’ diet caused the decrease of the ratio of n6/n3 in goat’s milk in the present study, which was in accordance with previous studies [16,41].

In addition, Table 1 and Table 4 were combined to explore if there was difference that existed in the FA profile between goat’s milk and Schizochytrium microalgae. In particular, C18:3n6, C20:1n9, C21:0, and C20:3n6 were only detected in the microalgae FA, and C18:1n7t and C18:2n6t were only detected in the goat’s milk FA. It is worth noting that C22:0 and C24:1, which did not exist in the control goat’s milk, were detected in all the test goat’s milk. Moreover, the content of C24:1 was increased with the additional amount of Schizochytrium microalgae powder. Therefore, the difference in the fatty acids’ profile between Schizochytrium microalgae and goat’s milk would provide a new way for distinguishing natural DHA dairy products from artificial DHA-adding dairy products.

### 4.4. Profile of Sn-2 Fatty Acids in Goat Milk

The synthesis of milk triglycerides is carried out by three different enzymes in the endoplasmic reticulum of mammary gland cells [44], and different appetencies of fatty acids for these enzymes can lead to various fatty acid compositions at triglyceride locations [45]. In recent decades, many studies have reported that microalgae can change the ruminant milk FA and improve the content of DHA in ruminant milk. To our knowledge, however, there was no study which studied the variation of sn-2 FA in ruminant milk after the dietary microalgae. In this study, the proportion of sn-2 DHA increased in the MM and HM goat’s milk in comparison with the control goat’s milk. By contrast, it decreased in the LM goat’s milk. The reason for this phenomenon was likely due to the low DHA level, which might have inhibited the activity of the related enzymes, and further affected the synthesis of triglycerides [44]. The sn-2 DHA was more advantageous in terms of absorption and utilization function compared with the random distribution of DHA [46]. Moreover, the absorption efficiency of sn-2 DHA was significantly higher than other derivatives, such as DHA diacylglycerol and DHA ethyl ester [47,48].

Most sn-2 MC-SFAs, such as sn-2 C10:0 and sn-2 C12:0, showed a rising tendency in the test goat’s milk. This was likely due to the MC-SFA, which had been previously hydrolyzed from TAG molecules. After that, the MC-SFA was transferred directly from the intestine to the blood and connected to the milk lipid [49]. MC-SFA had a positive effect on weight control and lipid metabolism when dairy products contained more MC-SFAs. Bohl, M (2017) reported that the lean body mass of obese adults increased after they ate higher MC-SFAs dairy products. Moreover, the fat accumulation, insulin resistance, blood pressure, and cholesterol concentration of these obese adults were restrained [50]. In addition, the proportion of sn-2 LC-SFA and MUFA decreased after the addition of microalgae into the fodder. However, the sn-2 C14:0 content increased significantly in the test goat milk. This phenomenon was different from the previous study which showed that the percentage of sn-2 LC-SFAs and MUFA all increased after the supplementation of flaxseeds into the sheep fodder [44], which was likely due to the existence of some differences in the fatty acid profiles of microalgae and flaxseeds.

## 5. Conclusions

In conclusion, the content of goat milk protein, SCC, production, and dry matter intake was not affected by the dietary Schizochytrium microalgae. Moreover, the milk fat content increased in LM goat’s milk compared with the control goat’s milk, and the lactose content showed a dropping trend in MM goat’s milk. The absolute concentration of DHA increased significantly in the experimental goat’s milk, with 29.485, 32.351, and 24.817 mg/100 g raw milk in the LM, MM, and HM goat milk, respectively. In addition, the sn-2 DHA content rose in MM and HM goat’s milk in comparison with the control goat’s milk. However, the proportion of sn-2 DHA decreased in LM goat’s milk. In addition, the content of major UFAs showed an increasing variation, except for the proportion of C18:1n9c which decreased in the test goat’s milk. The fatty acid profiles of goat ‘s milk and Schizochytrium microalgae was compared, and it was worth noting that C22:0 and C24:1 were only detected in the test goat’s milk. Therefore, the profile and content of the fatty acid and sn-2 fatty acid of goat’s milk were significantly affected by dietary Schizochytrium microalgae, especially in DHA. This result would promote the production of high DHA goat milk in practical production.

## Figures and Tables

**Table 1:** Ingredients and composition of the basal diet.

Item	Content (%)
Corn	48.10
Wheat bran	10.20
Soybean meal	13.60
Dry Alfalfa hay	22.00
NaHCO_3_	1.00
NaCl	0.60
Limestone	0.50
Premix	4.00
Total	100.00
Dry Matter (DM)	87.24
Crude Protein (CP)	13.62
Ether Extract (EE)	2.37
Neutral Detergent Fiber (NDF)	47.62
Acid Detergent Fiber (ADF)	22.73
Lignin	5.66

**Table 2:** Fatty acids composition of microalgae powder.

Item	Content (%)	Item	Content (%)
C4:0	0.16	C18:1n9c	7.7
C6:0	0.02	C18:2n6c	0.52
C8:0	0.88	C18:3n6	0.03
C10:0	0.03	C18:3n3	0.06
C11:0	0.01	C20:0	0.06
C12:0	0.06	C20:1n9	0.02
C14:0	0.35	C21:0	0.05
C14:1	0.02	C20:3n6	0.33
C15:0	0.04	C20:4n6	0.05
C15:1	0.19	C22:0	0.27
C16:0	6.74	C20:5n3	0.09
C16:1	0.16	C23:0	0.05
C17:0	0.03	C24:0	0.06
C17:1	0.12	C22:6n3	17.63
C18:0	0.67	C24:1	0.16

**Table 3:** Effect of supplementation microalgae on the content of routine milk and milk production of the test group.

Fatty Acids	Test Group				SEM	*p*-Value	Effect
	C	LM	MM	HM			
DMI (kg/day)	2.070	2.110	2.090	2.130	0.106	0.341	NS
Protein (%)	2.974	3.286	3.173	3.095	0.333	0.054	NS
Lipid (%)	3.067 ^a^	3.708 ^b^	3.357 ^ab^	3.017 ^a^	0.652	0.000	**
Lactose (%)	4.630 ^a^	4.713 ^a^	4.420 ^b^	4.745 ^a^	0.373	0.003	**
SCC (106/mL)	1.898	1.880	1.969	1.573	1.218	0.924	NS
Milk yield (kg/day)	1.633	1.806	1.502	1.802	0.714	0.307	NS
Concentration of DHA (mg/100 g raw material)	4.486 ^a^	29.485 ^b^	32.351 ^b^	24.817 ^b^	16.130	0.000	**

^a, b^ The letters indicate significant differences (*p* < 0.05) within a row; ** significant difference for which *p*-value < 0.01; NS significant difference for which *p*-value > 0.05. DMI = dry matter intake, SCC = Somatic cell counts.

**Table 4:** Effects for different dosage of microalgae supplementation on fatty acids (%).

Fatty Acids	Test Group				SEM	*p*-Value	Effect
	C	LM	MM	HM			
C4:0	0.805	0.828	0.918	0.939	0.255	0.300	NS
C6:0	1.815 ^a^	2.089 ^b^	2.013 ^b^	2.180 ^b^	0.307	0.010	*
C8:0	6.101 ^a^	3.424 ^b^	3.461 ^b^	3.742 ^b^	1.844	0.000	**
C10:0	9.466 ^a^	11.654 ^b^	11.154 ^b^	11.273 ^b^	1.670	0.002	**
C11:0	0.172	0.193	0.148	0.171	0.094	0.388	NS
C12:0	4.446 ^a^	5.601 ^b^	5.644 ^b^	5.318 ^b^	1.138	0.017	*
C14:0	10.452	11.027	11.157	10.401	1.252	0.117	NS
C14:1	0.235	0.189	0.189	0.209	0.089	0.444	NS
C15:0	1.006	1.054	0.962	1.039	0.225	0.467	NS
C15:1	0.221 ^a^	0.279 ^b^	0.275 ^b^	0.266 ^b^	0.060	0.042	*
C16:0	32.696	30.269	30.855	30.969	2.915	0.147	NS
C16:1	0.874	0.699	0.689	0.849	0.266	0.053	NS
C17:0	0.490 ^a^	0.585 ^b^	0.589 ^b^	0.553 ^b^	0.094	0.014	*
C17:1	0.231	0.192	0.186	0.203	0.067	0.275	NS
C18:0	5.894 ^ab^	6.559 ^b^	6.605 ^b^	5.126 ^a^	1.738	0.012	*
C18:1n7t	1.400 ^a^	3.686 ^b^	3.568 ^b^	5.315 ^c^	2.042	0.000	**
C18:1n9c	18.305 ^a^	15.274 ^b^	15.340 ^b^	14.821 ^b^	3.177	0.019	*
C18:2n6t	0.468 ^a^	0.596 ^b^	0.582 ^b^	0.540 ^ab^	0.120	0.016	*
C18:2n6c	3.932 ^b^	3.883 ^b^	3.355 ^a^	3.824 ^b^	0.762	0.001	**
C18:3n3	0.128 ^a^	0.176 ^b^	0.154 ^ab^	0.144 ^a^	0.043	0.006	**
C20:0	0.135 ^a^	0.154 ^ab^	0.173 ^b^	0.135 ^a^	0.038	0.001	**
C20:4n6	0.255	0.218	0.259	0.210	0.074	0.067	NS
C22:0	0.000 ^a^	0.061 ^b^	0.120 ^c^	0.109 ^c^	0.065	0.000	**
C20:5n3	0.163 ^a^	0.222 ^b^	0.256 ^b^	0.228 ^b^	0.080	0.011	*
C23:0	0.133 ^a^	0.254 ^bc^	0.308 ^c^	0.192 ^ab^	0.113	0.000	**
C24:0	0.069 ^a^	0.140 ^b^	0.189 ^c^	0.142 ^b^	0.070	0.000	**
C22:6n3	0.108 ^a^	0.623 ^b^	0.759 ^c^	0.585 ^b^	0.282	0.000	**
C24:1	0.000 ^a^	0.072 ^b^	0.094 ^b^	0.175 ^c^	0.103	0.000	**
SFA	73.680	73.892	74.294	72.290	3.724	0.311	NS
SC-SFA	2.620 ^a^	2.917 ^b^	2.931 ^b^	3.120 ^b^	0.382	0.005	**
MC-SFA	30.636	31.899	31.563	30.906	3.794	0.744	NS
LC-SFA	40.424	39.077	39.800	38.265	2.609	0.091	NS
MUFA	21.374	20.941	21.006	22.247	3.215	0.523	NS
PUFA	4.946 ^ab^	5.167 ^ab^	4.701 ^a^	5.463 ^b^	0.841	0.014	*
n3	0.399 ^a^	1.021 ^b^	1.170 ^c^	0.957 ^b^	0.325	0.000	**
n6	4.655 ^ab^	4.697 ^ab^	4.195 ^a^	4.916 ^b^	0.782	0.009	**
n6/n3	11.916 ^a^	4.905 ^b^	3.675 ^c^	5.419 ^b^	1.408	0.000	**

^a–c^ The letters indicate significant differences (*p* < 0.05) within a row; * significant difference for which *p*-value < 0.05; ** significant difference for which *p*-value < 0.01; NS significant difference for which *p*-value > 0.05. SFAs (saturated fatty acids) = ∑(C4:0, C6:0, C8:0, C10:0, C11:0, C12:0, C14:0, C15:0, C16:0, C17:0, C18:0, C20:0, C22:0, C23:0, C24:0); SC-SFA (short-chain saturated fatty acid) = ∑(C4:0, C6:0); MC-SFA (medium-chain saturated fatty acid) = ∑(C8:0, C10:0, C11:0, C12:0, C14:0); LC-SFA (long-chain saturated fatty acid) = ∑(C15:0, C16:0, C17:0, C18:0, C20:0, C22:0, C23:0, C24:0); MUFA (monounsaturated fatty acid) = ∑(C14:1, C15:1, C16:1, C17:1, C18:1n9c, C18:1n7t, C24:1); PUFA (polyunsaturated fatty acids) = ∑(C18:2n6c, C18:2n6t, C18:3n3, C20:4n6, C20:5n3, C22:6n6); n3 = ∑(C18:3n3, C20:5n3, and C22:6n3); n6 = ∑(C18:2n6c, C18:2n6t, C20:4n6).

**Table 5:** Effects for different dosage of microalgae supplementation on sn-2 fatty acids (%).

Fatty Acids	Test Group				SEM	*p*-Value	Effect
	C	LM	MM	HM			
C4:0	0.097	0.053	0.086	0.123	0.051	0.466	NS
C6:0	0.105	0.052	0.086	0.110	0.036	0.174	NS
C8:0	0.483 ^b^	0.212 ^a^	0.301 ^a^	0.364 ^ab^	0.124	0.018	*
C10:0	1.462 ^a^	4.043 ^b^	6.648 ^c^	5.565 ^c^	2.117	0.000	**
C11:0	0.154	0.177	0.123	0.161	0.030	0.151	NS
C12:0	3.326 ^a^	6.280 ^b^	6.262 ^b^	4.691 ^c^	1.338	0.000	**
C14:0	14.887 ^a^	19.282 ^b^	18.612 ^b^	14.947 ^a^	2.28	0.001	**
C14:1	0.316 ^a^	0.470^b^	0.128 ^c^	0.156 ^c^	0.152	0.000	**
C15:0	1.016	1.187	1.071	0.999	0.101	0.066	NS
C15:1	8.678	3.704	3.909	6.676	3.179	0.158	NS
C16:0	40.235	36.210	36.128	35.492	2.582	0.064	NS
C16:1	0.673	0.428	0.607	0.666	0.190	0.397	NS
C17:0	0.454	0.500	0.572	0.471	0.080	0.308	NS
C17:1	0.317 ^c^	0.128 ^a^	0.236 ^b^	0.254 ^b^	0.075	0.000	**
C18:0	10.397	7.953	8.954	10.060	1.664	0.279	NS
C18:1n7t	1.429 ^a^	2.611 ^ab^	2.606 ^ab^	3.945 ^b^	1.133	0.024	*
C18:1n9c	11.392 ^a^	7.668 ^b^	9.568 ^b^	10.357 ^ab^	2.197	0.027	*
C18:2n6t	0.191	0.252	0.358	0.281	0.108	0.321	NS
C18:2n6c	2.975	1.755	1.531	2.257	0.852	0.155	NS
C18:3n3	0.103 ^a^	0.053 ^b^	0.029 ^b^	0.033 ^b^	0.033	0.001	**
C20:0	0.266 ^a^	0.361 ^b^	0.322 ^b^	0.348 ^b^	0.045	0.018	*
C20:4n6	0.630 ^a^	0.463 ^a^	0.973 ^b^	0.614 ^a^	0.223	0.006	**
C22:0	0.000 ^a^	0.000 ^a^	0.017 ^a^	0.063 ^b^	0.029	0.001	**
C20:5n3	0.073 ^a^	0.098 ^ab^	0.138 ^b^	0.139 ^b^	0.038	0.048	*
C23:0	0.147 ^a^	0.164 ^a^	0.148 ^a^	0.502 ^b^	0.168	0.000	**
C24:0	0.027	0.077	0.046	0.074	0.029	0.074	NS
C22:6n3	0.166 ^a^	0.104 ^b^	0.354 ^c^	0.429 ^c^	0.056	0.032	*
C24:1	0.000 ^a^	0.065 ^c^	0.024 ^b^	0.04 ^b^	0.027	0.001	**
SFA	73.057 ^a^	76.549 ^ab^	79.374 ^b^	73.968 ^a^	3.268	0.044	*
SC-SFA	0.203	0.105	0.171	0.233	0.079	0.234	NS
MC-SFA	20.312 ^a^	29.993 ^c^	31.945 ^c^	25.727 ^b^	4.900	0.000	**
LC-SFA	52.542 ^a^	46.451 ^b^	47.258 ^b^	48.008 ^b^	2.949	0.017	*
MUFA	22.97 ^b^	15.113 ^a^	17.409 ^a^	22.484 ^b^	3.984	0.007	**
PUFA	3.972	2.686	3.053	3.364	0.822	0.288	NS
n3	0.176	0.216	0.191	0.212	0.042	0.667	NS
n6	3.796	2.470	2.863	3.152	0.846	0.292	NS
n6/n3	22.752 ^a^	11.727 ^b^	15.011 ^b^	15.239 ^b^	3.424	0.027	*

^a–c^ The letters indicate significant differences (*p* < 0.05) within a row; * significant difference for which *p*-value < 0.05; ** significant difference for which *p*-value < 0.01; NS significant difference for which *p*-value > 0.05. SFAs (saturated fatty acids) = ∑(C4:0, C6:0, C8:0, C10:0, C11:0, C12:0, C14:0, C15:0, C16:0, C17:0, C18:0, C20:0, C22:0, C23:0, C24:0); SC-SFA (short-chain saturated fatty acid) = ∑(C4:0, C6:0); MC-SFA (medium-chain saturated fatty acid) = ∑(C8:0, C10:0, C11:0, C12:0, C14:0); LC-SFA (long-chain saturated fatty acid) = ∑(C15:0, C16:0, C17:0, C18:0, C20:0, C22:0, C23:0, C24:0); MUFA (monounsaturated fatty acid) = ∑(C14:1, C15:1, C16:1, C17:1, C18:1n9c, C18:1n7t, C24:1); PUFA (polyunsaturated fatty acids) = ∑(C18:2n6c, C18:2n6t, C18:3n3, C20:4n6, C20:5n3, C22:6n6); n3 = ∑(C18:3n3, C20:5n3, and C22:6n3); n6 = ∑(C18:2n6c, C18:2n6t, C20:4n6).

**Table 6:** Comparison of the content and absolute concentration of DHA in ruminant milk.

Reference ^a^	Species	Feeding Time	Addition of Microalgae (g)	Addition of DHA (g)	Content (%)	Absolute Concentration (mg/100 g raw milk)	Increase Ratio of DHA (%)
Franklin (1999) [12]	cow (*n* = 30)	6 weeks	910	-	0.76	-	760
PAPADOPOULOS (2002) [29]	ewes (*n* = 32)	6 weeks	23.5	-	0.43	-	460
		6 weeks	47	-	0.69	-	430
		6 weeks	94	-	1.24	-	690
Moate (2013) [24]	cow (*n* = 32)	30 days	125	25.00	0.36	-	1240
		30 days	250	50.00	0.60	-	500
		30 days	375	75.00	0.91	-	900
Vahmani (2013) [13]	cow (*n* = 48)	125 days	200	48.50	0.20	-	1417
Póti (2015) [15]	goat (*n* = 20)	17 days	30	-	0.04	-	900
	cow (*n* = 16)	17 days	266.4	-	0.02	-	100
Moran (2018) [38]	cow (*n* = 12)	84 days	100	16.00	0.10	-	100
Sinedino (2017) [39]	cow (*n* = 366)	174 days	100	10.00	0.24	-	100
Pajor (2019) [16]	goat (*n* = 10)	31 days	15	2.00	0.40	15.70	242
Till (2019) [40]	cow (*n* = 60)	14 weeks	100	-	0.22	-	400
Liu (2020) [14]	cow (*n* = 36)	60 days	170	30.00	0.37	-	450
		60 days	255	45.00	0.53	-	370
Mavrommatis (2020) [41]	goat (*n* = 24)	74 days	20	4.16	0.70	-	530
		74 days	40	8.44	1.30	-	700
		74 days	60	8.20	1.51	-	1300
This study	goat (*n* = 120)	65 days	15	2.64	0.62	29.49	1510
		65 days	25	4.41	0.76	32.35	477
		65 days	35	6.17	0.59	24.88	603

^a^: Reference: (Moate et al., 2013, Vahmani et al., 2013b, Franklin et al., 1999, Papadopoulos et al., 2002, Póti et al., 2015, Moran et al., 2018, Sinedino et al., 2017, Pajor et al., 2019, Till et al., 2019, Liu et al., 2020, Mavrommatis and Tsiplakou, 2020).

## Data Availability

Data is contained within the article.
